# Amperometric Immunosensor Based on a Protein A/Deposited Gold Nanocrystals Modified Electrode for Carbofuran Detection

**DOI:** 10.3390/s111211679

**Published:** 2011-12-15

**Authors:** Xia Sun, Ying Zhu, Xiangyou Wang

**Affiliations:** School of Agriculture and Food Engineering, Shandong University of Technology, No.12, Zhangzhou Road, Zibo 255049, China; E-Mails: sunxia2151@sina.com (X.S.); zy_881120@126.com (Y.Z.)

**Keywords:** amperometric immunosensor, deposited gold nanocrystals, protein A, carbofuran

## Abstract

In this paper, an amperometric immunosensor modified with protein A/deposited gold nanocrystals (DpAu) was developed for the ultrasensitive detection of carbofuran residues. First, DpAu were electrodeposited onto the Au electrode surface to absorb protein A (PA) and improve the electrode conductivity. Then PA was dropped onto the surface of DpAu film, used for binding antibody Fc fragments. Next, anti-carbofuran monoclonal antibody was immobilized on the PA modified electrode. Finally, bovine serum albumin (BSA) was employed to block the possible remaining active sites avoiding any nonspecific adsorption. The fabrication procedure of the immunosensor was characterized by electrochemical impedance spectroscopy (EIS) and cyclic voltammetry (CV), respectively. With the excellent electroconductivity of DpAu and the PA’s oriented immobilization of antibodies, a highly efficient immuno-reaction and detection sensitivity could be achieved. The influences of the electrodeposition time of DpAu, pH of the detection solution and incubation time on the current response of the fabricated immunosensor were investigated. Under optimized conditions, the current response was proportional to the concentration of carbofuran which ranged from 1 to 100 ng/mL and 100 ng/mL to 100 μg/mL. The detection limit was 0.1924 ng/mL. The proposed carbofuran immnuosensor exhibited high specificity, reproducibility, stability and regeneration performance, which may open a new door for ultrasensitive detection of carbofuran residues in vegetables and fruits.

## 1. Introduction

Carbofuran (2,3-dihydro-2,2-dimethylbenzofuran-7-yl methylcarbamate) is a broad-spectrum insecticide widely used in agriculture to control pests in horticultural crops to enhance production and control of insect-borne diseases. The presence of carbofuran in food has received worldwide attention because of its relatively high solubility of 700 mg/L in water at 25 °C and the fact it is a systemic insecticide, which can be absorbed by roots, steams and leaves and thus transmit and translocate in ther plant. Carbofuran can also cause acute toxicity to human through cholinesterase inhibition [1,2].

Monitoring of pesticide residues in vegetables for evaluation of vegetable quality so as to avoid possible risks to human health is a priority objective [3]. Current analytical methods for carbofuran detection involving gas chromatography (GC) and high performance liquid chromatography (HPLC) with post-column derivatization are sensitive and reliable [4,5], but these methods require expensive instruments, skilled analysts and involve time-consuming sample preparation steps [6]. Therefore, there is a growing demand for more rapid and economical methods for detecting pesticide residues.

It has been reported that biosensor measurements are a good method for rapid detection of pesticide residues [3]. Immunosensors, which combine the selectivity provided by immunological interactions, are also being proposed and proving to be powerful analytical devices for the monitoring of organic pollutants in food and the environment [7,8]. The immobilization of biomolecules on the electrode is the most important factor in generating rapid response and in fabricating high selective biosensor.

In order to develop electrochemical immunosensor technology, the investigation of novel composite materials has attracted widespread attention, due to their excellent electronic transport properties, good biocompatibility, satisfying stability and so on [9,10]. Gold nanoparticles (GNPs) have been widely used for immobilization of biomolecules due to their large specific surface area, high surface free energy and biocompatibility. GNPs can adsorb biomolecules and play an important role in the immobilization of biomolecules for biosensor construction [11]. So far, GNPs have been widely applied in the biosensors for detection of pesticide residues [12–14]. However, most biosensors based on GNPs were modified with gold nanoparticles colloids, which involved time-consuming preparation steps and low adsorption. Therefore, deposited gold nanocrystals (DpAu) were studied because it could provide a stable surface for the immobilization of biomolecules [15,16]. Morver, the DpAu film could be controled through the concentrtion of gold chloride tetrahydrate (HAuCl_4_) solution and the electrodeposition time, which is more convenient than GNPs film.

Protein A has been commonly used as a binding material to orientedly immobilize antibodies because it can specially bind the Fc fragment of the antibody molecules. The antibody after oriented immobilization is favorable for antigen accessible [17–19]. As protein A can adsorb firmly onto gold surface, the traditional antibody immobilizations using PA tend to assemble protein A directly onto the gold-derivatized sensor transducers [20]. Such a convenient PA-based immobilization procedure might cause some problems associated with low loading amount and gold-induced denaturation of PA, which results in low antibody-binding capacities and very few reuses of the sensors. In order to overcome these shortages, DpAu with high protein-loading capacity have been used to increase the amount of immobilized biomolecules. For example, Wang *et al*., have investigated a PA-based immobilization methodology for antibodies [21]. In recent years, PA has been used in the biosensors for the determination of phenylurea herbicide diuron, the herbicide simazine, and penicillin residues in milk [12,22,23].

As mentioned above, using DpAu can immobilize PA efficiently as well as improve the electrode conductivity. Using PA can immobilize antibody orientedly. However, most researchers have utilized GNPs instead of DpAu. DpAu could provide a porous and stable surface for the immobilization of PA. To the best of our knowledge, immonosensors based on strong reaction between DpAu and PA for the detection of pesticide residues have not been reported. In this work, we developed an amperometric immunosensor modified with PA/DpAu for the detection of carbofuran. Optimal conditions of the described immunosensor are investigated in detail.

## 2. Experimental

### 2.1. Materials

Anti-carbofuran monoclonal antibody, carbofuran, protein A and bovine serum albumin (BSA) were all purchased from Sigma. HAuCl_4_ was from Shanghai Sinopharm Chemical Reagent Co. Ltd., China. Carbofuran was standard product and other reagents were of analytical grade and distilled water was used throughout the experiments. Anti-carbofuran monoclonal antibody was dissolved with 0.01 M phosphate buffer solution (PBS, pH 7.4) processed by high-pressure sterilization and stored at 4 °C. A PBS (0.1 M, pH 7.4) containing 5 mM [Fe(CN)_6_]^3−/4−^ and 0.1 M KCl was used as the detection solution.

### 2.2. Apparatus

Cyclic voltammetry (CV) and the electrochemical impedance spectroscopy (EIS) measurements were performed with CHI 650D electrochemical workstation (Shanghai Chenhua Co., China). All expriments were performed with a conventional three-electrode system. The modified gold electrode (d = 1 mm) as the working electrode, a saturated calomel electrode (SCE) and platinum electrode were used as reference and auxiliary electrodes, respectively. The morphology of GNPs and DpAu film was studied by means of scanning electron microscopy (SEM, S-3000N, Hitachi, Japan).

### 2.3. Fabrication of Immunosensor

Gold electrodes with 1 mm diameter were polished carefully with 0.5 μm and 30 nm alumina powder. Then they were sonicated with piranha solution (a 1:3 mixture of 30% H_2_O_2_/concentrated H_2_SO_4_) for 10 min, and washed with distilled water. Next, they were immersed successively in 6 M HNO_3_, absolute ethanol and distilled water in an ultrasonic bath for 5 min to remove any physically absorbed substances. Before modification, the bare gold electrodes were scanned in 0.5 mM H_2_SO_4_ between −0.3 to 1.5 V at a scan rate of 0.05 V/s until a reproducible cyclic voltammogram (CV) was obtained. After that, the cleaned electrode was rinsed with distilled water, and then was dried in a stream of nitrogen.

**Figure 1.**
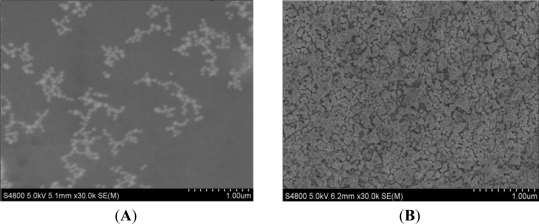
SEM images of GNPs film (**A**) and DpAu film (**B**) on Au electrode.

**Scheme 1.**
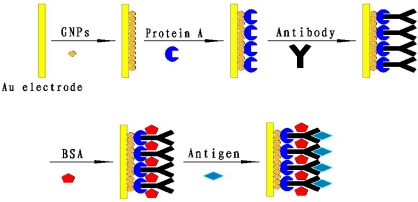
Schematic illustration of the stepwise immunosensor fabrication process.

The electrode was modified immediately after the cleaning step. DpAu was electrodeposited onto the pretreated gold electrode at −0.2 V for 100 s in a solution containing 2 mg/mL HAuCl_4_ and 0.1 M KNO_3_. SEM images of GNPs and DpAu are shown in Figure 1(A,B), respectively. From Figure 1(A) we can see that there are some small diameter GNPs on the bare Au electrode but the distribution is uneven. Compared with the GNPs [Figure 1(A)], the SEM image showed that the DpAu formed a uniformer and porous film onto the surface of electrode [Figure 1(B)], it also showed that DpAu was electrodeposited onto the gold electrode successfully. After rinsing thoroughly with distilled water and dried in a stream of nitrogen. Then 5 μL of 0.5 mg/mL protein A was dropped on the DpAu/Au modified electrode at room temperature for 1 h and then the electrodes was immersed in 5 μg/mL anti-carbofuran antibody and kept for at least 12 h at 4 °C. Finally, the electrode was incubated with 2.5% BSA at room temperature for 1 h in order to block nonspecific binding sites. The resulted immunosensor was stored above the 0.1 M PBS at 4 °C when not in use. The schematic illustration of the fabrication process was shown in Scheme 1.

### 2.4. Experimental Method

The carbofuran detection was based on relative change in current response (I% = (I_0_ − I_1_)/I_0_) where I_0_ and I_1_ were the cathodic peak currents of the CVs before and after the immunosensor’s reaction to the antigen, respectively.

### 2.5. Regeneration of the Immunosensor

After the immunization, the electrodes were immerged in 0.1 M citrate buffer solution (CBS, pH 2.7) for 5 min, in order to separate antigen-antibody complex from the PA surface, and then immobilized anti-carbofuran refering to the above steps for carbofuran detection.

## 3. Results and Discussion

### 3.1. Characteristics of the Electrochemistry on Electrode Surface

**Figure 2.**
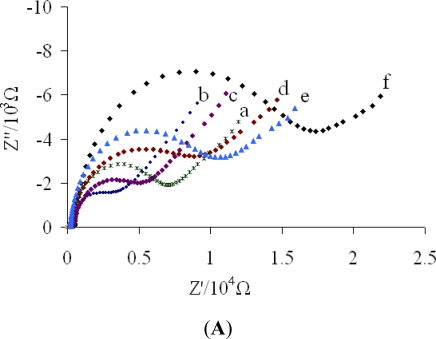

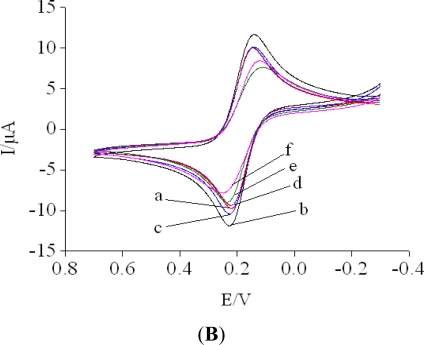
EIS (**A**) and CVs (**B**) of different electrodes in 5 mM [Fe(CN)_6_]^3−/4−^: (a) bare Au electrode; (b) DpAu/Au; (c) PA/DpAu/Au; (d) anti-carbofuran/PA/DpAu/Au; (e) BSA/anti-carbofuran/PA/DpAu/Au; (f) carbofuran/BSA/anti-carbofuran/PA/DpAu/Au.

The EIS of different electrodes are shown in Figure 2(A). DpAu with large specific area and high electrical conductivity could improve the conductivity of the electrode, thus after DpAu were deposited onto Au electrode, the charge transfer resistance (Rct) decreased (curve b), demonstrating that DpAu have been successfully assembled on the bare Au electrode (curve a), which is consitent with previous reports [24]. When PA was immobilized on the electrode surface, Rct obviously increased (curve c), which was ascribed the inhibition effect of PA biomacromolecules for electron transfer [21]. Then anti-carbofuran was covalently immobilized on the PA/DpAu/Au, the Rct obviously increased further (curve d). The reason was that anti-carbofuran forms an insulating layer on the electrode surface, leading to a higher electron transfer resistance. A further increase was noticed (curve e) when the anti-carbofuran/PA/DpAu/Au was blocked with BSA. The explanation was that BSA can block possible remaining active sites and further hinder the electron transfer, which could clearly confirm that BSA was successfully immobilized on the electrode. After the immunosensor was incubated with 50 ng/mL carbofuran solution, a further increase in Rct was noted (curve f). This was because the formed immunocomplex on the electrode surface acted as an inert block layer. Thus hindered the diffusion of electron towards the electrode surface, which was in good agreement with the previous reports [25].

**Figure 3.**
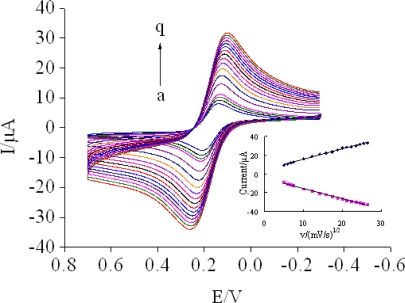
CVs of the modified elecrode at different scan rates (from a to q): 25, 35, 45, 55, 100, 150, 200, 250, 300, 350, 400, 450, 500, 550, 600, 650, 700 mV/s in 5 mM [Fe(CN)_6_]^3−/4−^.

In addition, cyclic voltammograms (CVs) were also used to monitor the fabrication process. Figure 2(B) showed the CV of different electrodes, in agreement with EIS results. Figure 3 shows the CVs of the prepared immunosensor in 5 mM [Fe(CN)_6_]^3−/4−^ at different scan rates. It could be found that both the anodic and cathodic peak currents increased linearly with v^1/2^ in the ranges of 25–700 mV/s. The regression equations of the two straight lines are as follows: Ipa(μA) = −4.851 − 1.0815v^1/2^ (mV/s) (R^2^ = 0.9959), Ipc(μA) = 4.4903 + 1.1101v^1/2^ (mV/s) (R^2^ = 0.9959). This result indicated that the electrochemical process was a diffusion-controlled reaction [26].

### 3.2. Optimization Conditions for Immunoassay

**Figure 4.**
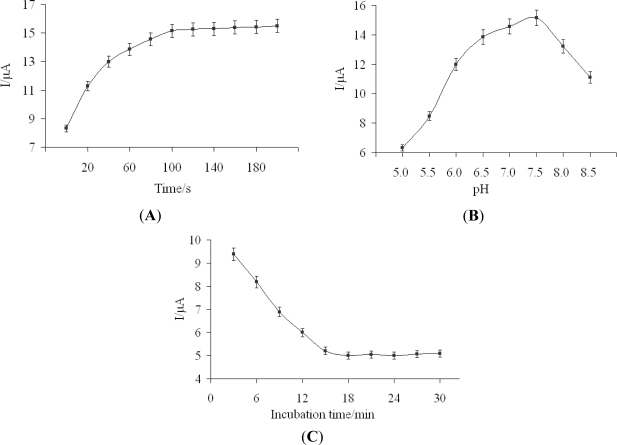
Effect of the electrodeposition time of DpAu (**A**), the pH of 5 mM [Fe(CN)_6_]^3−/4−^ (**B**) and the incubation time (**C**) on the immunosensor response.

The electrodeposition of DpAu onto Au electrode would improve the conductivity of the electrode and increase the absorbance of PA, so the electrodeposition time of DpAu greatly affected the analytical performance of the proposed immunosensor. As shown in Figure 4(A), the response current increased with increasing electrodeposition time from 20 s to 100 s. However, when the electrodeposition times were longer than 100 s, the current response tends to be steady. The reason would be that thick DpAu layer hindered the electron transfer. Considering the requirement of our study and economic situation, 100 s was chosen as the optimal electrodeposition time.

The pH of the detection solution could influence the activity of the protein activity and the antigen-antibody reaction, thus the effect of pH values from 5.0 to 8.5 on the immunosensor performance was investigated in this work. As shown in Figure 4(B), current response increased with the solution pH increasing from 5.0 to 7.5, reaching a maximum current response at pH 7.5. However, when the pH was more than 7.5, the current response decreased. Thus, pH 7.5 was chosen as the optimal value and used throughout the experiment.

The incubation time also influenced the immunosensor response for carbofuran detection. The immunosensor was incubated with 50 ng/mL carbofuran solution for different times at room temperature. Figure 4(C) showed that the current response decreased with increasing the incubation time at plateau at 15 min. Longer incubation time did not cause further decreases of the response current, indicating that the specific binding of antigen and antibody has reached equilibrium. As a result, the optimum incubation period was set at 15 min for the incubation steps in this study.

### 3.3. Detection of Carbofuran Using the Immunosensor

**Figure 5.**
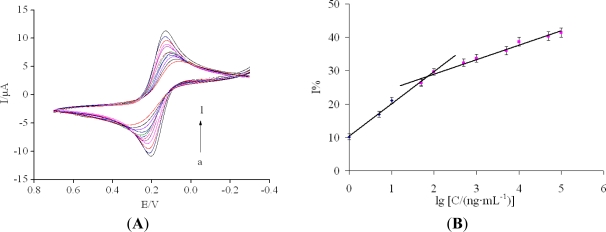
(**A**) The CVs of the immunosensor after incubation in different concentrations of carbofuran standard solution (from a to l): 0, 1, 5, 10, 50, 100, 500, 1.0 × 10^3^, 5.0 × 10^3^, 1.0 × 10^4^, 5.0 × 10^4^, 1.0 × 10^5^ ng/mL under the optimal conditions; (**B**) The calibration curve of the relative change of current response of the proposed immunosensor *versus* the logarithm of carbofuran concentration.

Under the optimized experimental conditions, the developed immunosensor was used to detect the carbofuran solution of different concentrations. Figure 5(A) shows the CVs of the immunosensor incubated with different carbofuran concentrations. It was found that the current response decreased with carbofuran concentration. This may be due to more carbofuran binding to the immobilized antibodies at higher carbofuran concentrations, which acts as a definite kinetic barrier for the electron transfer. A linear relationship between the relative change in the current response and logarithm of carbofuran solution was obtained from 1 to 100 ng/mL with a regression equation: I% = 10.426 + 9.6704 lgC (ng/mL) (R^2^ = 0.9903) and another linear relationship from 100 ng/mL to 100 μg/mL with a regression equation: I% = 20.263 + 4.3357 lgC (ng/mL) (R^2^ = 0.9796) [Figure 5(B)]. We calculated the detection limit of 0.1924 ng/mL at a signal-to-noise ratio of 3 (S/N = 3) between the detection signal of low concentration samples and the noise of blank samples.

The performance of the BSA/anti-carbofuran/PA/DpAu/Au sensor was compared with other reported immunosensors for the detection of carbofuran previously. As shown in Table 1, compared with other methods, the immunosensor has a relative large linear range and lower detection limit.

### 3.4. Specificity, Reproducibility, Stability and Regeneration of the Immunosensor

**Figure 6.**
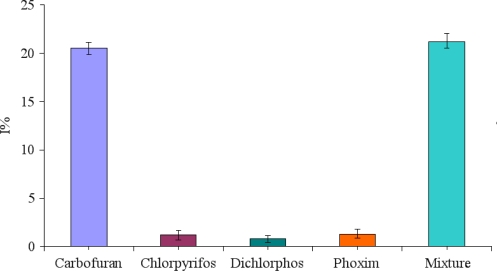
Relative change of current response of the immunsensor to 50 ng/mL carbofuran, 50 ng/mL chlorpyrifos, 50 ng/mL dichlorphos, 50 ng/mL phoxim and the mixture containing 50 ng/mL carbofuran, 50 ng/mL chlorpyrifos, 50 ng/mL dichlorphos and 50 ng/mL phoxim.

To investigate the specificity of the immunosensor, we detected the current response of the immunosensor to carbofuran, other small molecules (chlorpyrifos, dichlorphos, phoxim) commonly present in real samples with same concentration of 50 ng/mL and their mixture. The relative changes of current response of the proposed immunosensor are shown in Figure 6. It can be seen that no significant changes are obtained for these interferences. It means that the developed immunosensor holds a high degree of selectivity for carbofuran detection.

The reproducibility of the immunosensor was estimated by determining 50 ng/mL carbofuran solutions with four immunosensors. Four electrodes exhibited similar current response and the relative standard deviation (RSD) was 3.6%. The results showed that the proposed immunosensor can be used repeatedly with an acceptable reproducibility.

**Figure 7.**
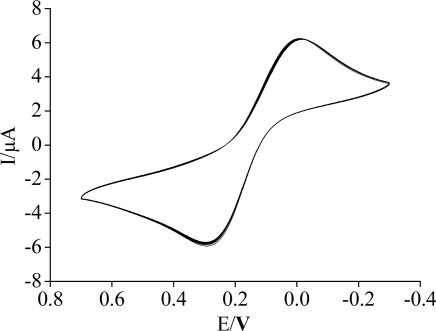
30 cycles CVs of the immunosensor in 5 mM [Fe(CN)_6_]^3−/4−^.

Under the optimal conditions, the immunosensor was measured by CV for 30 cycle successive scan, and a 2.4% deviation of the initial response was observed (Figure 7). The prepared immunosensors were suspended over the PBS at 4 °C for 2 weeks, and measured the current response every day. The immunosensors retained over 91% of their initial responses, indicating acceptable stability.

**Figure 8.**
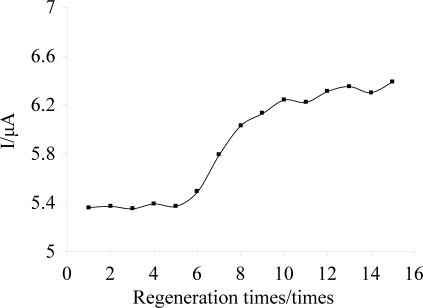
Regeneration performance of the immunosensor.

Good regeneration performance is an important index for the popularization and application of an immunosensor. Figure 8 shows the current responses of the immunosensor in 50 ng/mL carbofuran solutions after processing by CBS and immobilized anti-carbofuran. As shown in Figure 8, after regenerating six times, the current response increased sharply. This is likely because PA can gradually shell off during continuous cleaning with the increase of regeneration times. Therefore, it leads to the anti-carbofuran antidody cannot immobilize onto the DpAu surface orientedly, and it furtherly affects the binding activities between antibody and antigen [31]. The results showed that the immunosensor had a good regeneration performance and could regenerate 6 times.

### 3.5. Real Sample Analysis

In order to evaluate the feasibility of the proposed immunosensor for vegetables analysis, Chinese chive and celery cabbage samples were examined. A series of samples was prepared by adding carbofuran of different concentrations to these vegetable samples. The results were exhibited in Table 2 and the RSD between 2.92% and 4.36% were obtained. The recovery was in the range 94.0%–106.1%, suggesting that the proposed immunosensor could be feasible for the direct analysis of carbofuran in real samples.

## 4. Conclusions

In this work, we introduced a strategy for preparing a new label-free amperometric BSA/anti-carbofuran/PA/DpAu/Au immunosensor, which successfully immobilized the anti-carbofuran antibody on the modified electrode surface for the detection of carbofuran. Compared with GNPs, the DpAu film can immobilize PA efficiently, because it could overcome the low loading amount and gold-induced denaturation of PA, save the time and steps of preparation, and improve the electrode conductivity. Besides, due to PA’s specially binding ability of the Fc fragment of the antibody molecules, the application of PA could improves the capacity of antibody, thus enhance the detection sensitivity. With this strategy, a detection limit of 0.1924 ng/mL was achieved for carbofuran. Moreover, the developed immunosensor showed high specificity, reproducibility, stability and regeneration performance, which may find potential applications for the detection of other pesticides or compounds.

## Figures and Tables

**Table 1.** Comparison with other reported immunosensors for the detection of carbofuran.

**Electrode**	**Liner range (ng/mL)**	**Detection limit (ng/mL)**	**References**
AChE/PAMAM-Au/CNTs/GCE	1–20	0.89	[27]
AuNP/AChE/Au	-	7.293	[28]
HRP/Ab/GNPs/L-cysteine/Au	40–140	40	[29]
Carbofuran/Ab/SiSG/GCE	1–10^5^	0.33	[30]
BSA/Ab/PA/DpAu/Au	1–10^5^	0.1924	This work

**Table 2.** The recovery of the proposed immunosensor in real samples.

**Sample**	**Added (ng/mL)**	**Found (ng/mL)**	**RSD (%) (n = 3)**	**Recovery (%)**
Chinese chive	10	10.43	4.18	104.3
	1.0 × 10^2^	0.97 × 10^2^	3.55	97.0
	1.0 × 10^3^	0.94 × 10^3^	3.81	94.0
Celery cabbage	10.0	10.61	4.36	106.1
	1.0 × 10^2^	0.96 × 10^2^	3.37	96.0
	1.0 × 10^3^	1.03 × 10^3^	2.92	103.0
